# Deciphering the prognostic role of endoplasmic reticulum stress in lung adenocarcinoma: integrating prognostic prediction and immunotherapy strategies

**DOI:** 10.1007/s10238-024-01439-4

**Published:** 2024-07-25

**Authors:** Bing Wen, Pengpeng Zhang, Jiping Xie, Zhaokai Zhou, Ge Zhang, Lianmin Zhang, Zhenfa Zhang

**Affiliations:** 1 Department of Lung Cancer, Tianjin Lung Cancer Center, National Clinical Research Center for Cancer, Key Laboratory of Cancer Prevention and Therapy, Tianjin’s Clinical Research Center for Cancer, Tianjin Medical University Cancer Institute and Hospital, Tianjin, China; 2 Department of Cardiothoracic Surgery, The Second People’s Hospital of Yibin, Yibin, Sichuan China; 3 Department of Urology, The First Affiliated Hospital of Zhengzhou University, Zhengzhou, China; 4 Department of Cardiology, The First Affiliated Hospital of Zhengzhou University, Zhengzhou, China

**Keywords:** Lung adenocarcinoma, Endoplasmic reticulum stress, TME, Prognosis,signature

## Abstract

Endoplasmic reticulum stress (ERS) is a critical factor influencing lung adenocarcinoma (LUAD) progression and patient outcomes. In this study, we analyzed gene expression data from LUAD samples sourced from The Cancer Genomic Atlas and Gene Expression Omnibus databases. Utilizing advanced statistical methods including LASSO and Cox regression, we developed a ERS-associated signature (ERAS) based on ten ERS-related genes. This model stratified patients into high- and low-risk groups, with the high-risk group exhibiting decreased survival rates, elevated tumor mutational burden, and heightened chemotherapy sensitivity. Additionally, we observed lower immune and ESTIMATE scores in the high-ERAS group, indicating a potentially compromised immune response. Experimental validation through quantitative real-time polymerase chain reaction confirmed the utility of our model. Furthermore, we constructed a nomogram to predict 1-, 3-, and 5-year survival rates, providing clinicians with a valuable tool for personalized patient management. In conclusion, our study demonstrates the efficacy of the ERAS in identifying high-ERAS LUAD patients, offering promising implications for improved prognostication and treatment strategies.

**Supplementary Information:**

The online version contains supplementary material available at 10.1007/s10238-024-01439-4.

## Introduction

Lung cancer (LC) is considered one of the most prevalent malignant tumors worldwide, posing a significant threat to human life and health [1]. It is commonly categorized into two main types: small cell lung cancer and non-small cell lung cancer (NSCLC), with the latter comprising the majority (85%). Among NSCLC subtypes, lung adenocarcinoma (LUAD) stands as the predominant variant, accounting for approximately half of all lung cancer cases [2]. Scientists and health-care professionals have made substantial strides in developing various therapeutic approaches for lung cancer, which can be tailored based on the patient’s pathological type and staging. Despite notable advancements in technology in recent years, the 5-year survival rate for lung cancer remains less than optimistic [3]. However, if physicians could accurately predict a patient’s prognosis before treatment, there would be an opportunity to devise a treatment plan that best aligns with their specific condition, leading to personalized and targeted therapies [4, 5]. Therefore, there is an urgent need for a more precise and effective prognostic tool to guide clinical practitioners in their decision-making process.

The endoplasmic reticulum (ER) is a crucial cellular organelle involved in protein synthesis, folding, and repair. When cells are exposed to various internal and external stimuli such as oxidative stress, calcium imbalance, nutrient deficiency, and drug toxicity, the ER’s normal functioning is disrupted, leading to the occurrence of ER stress (ERS) as a mechanism to restore ER functionality [6, 7]. Cancer cells often reside in highly hostile environments, characterized by nutrient deprivation, hypoxia, drug toxicity, and other challenges, all of which can induce ERS [8]. The role of ERS in cancer can be dualistic. On the one hand, ERS can enhance cancer cells’ adaptability to adverse conditions by activating stress response pathways. On the other hand, excessive ERS can lead to apoptosis in cancer cells, thereby inhibiting tumor growth to a certain extent [9]. The previous studies have investigated the role of ERS in LUAD. Shu et al. identified a correlation between ERS-related genes and the prognosis of LUAD, culminating in the development of a predictive model for LUAD based on 18 pertinent genes [10]. Xin et al. pioneered a signature utilizing five lncRNAs linked to ERS, unveiling that patients classified as high-risk exhibited diminished survival rates and heightened responsiveness to immunotherapy [11]. Based on the above, we believe that ERS-related genes hold potential as predictive indicators and therapeutic targets, warranting further exploration of the relationship between ERS and LUAD.

In this study, our aim is to identify ERS-related genes that impact the prognosis of LUAD. Furthermore, we will construct a novel signature based on these ERS-related genes to predict LUAD outcomes. Additionally, we will explore the application of this signature in guiding immunotherapy and chemotherapy drug selection, providing effective guidance for the diagnosis and treatment of LUAD.

## Method

### Dataset source

The Cancer Genome Atlas (TCGA) repository served as a source for a substantial collection of data, encompassing RNA sequencing, methylation, CNV, mutation, and clinical information pertinent to LUAD. This database provided a comprehensive dataset for our study. Concurrently, the scRNA-seq dataset GSE189357 [12], featuring samples from nine treatment-naïve LUAD patients (TD1-9), was accessed from the Gene Expression Omnibus (GEO). These samples were derived from patients diagnosed with AIS, MIA, and IAC. Additionally, seven other LUAD-related datasets were procured from GEO, each containing vital clinical survival data. These datasets, namely, GSE13213 [13], GSE26939 [14], GSE29016 [15], GSE30219 [16], GSE31210 [17], GSE42127 [18], and GSE68465 [19], encompassed a wide range of patient numbers and were integral for validating the developed model. To achieve consistency and comparability across the datasets, gene expression data were converted to the TPM format. To address and rectify batch effects, the “combat” feature within the “sva” R package [20] was applied. Moreover, a uniform logarithmic transformation was applied to all sequencing data acquired from the TCGA and GEO databases, ensuring a standardized format for subsequent analysis.

### The detailed steps of the single-cell analysis process

Utilizing Seurat the R package (version 4.2.0), the initial gene expression matrix underwent processing [21]. Criteria for gene inclusion mandated expression in a minimum of 10 cells per sample. Cells not meeting quality standards—those expressing either over 6000 or under 200 genes, or those with mitochondrial genome UMIs exceeding 10%—were excluded from the analysis. This filtration ensured the selection of high-quality cells for further study. The transcriptomic expression data of these selected cells were then integrated using the Harmony R package. For the identification of highly variable genes, principal component analysis (PCA) was employed. The most significant 30 principal components (PCs) were then utilized for UMAP dimension reduction. This process facilitated the visualization of gene expression across various cell subpopulations. Differential gene expression (DEGs) within these subpopulations was ascertained using the “FindAllMarker” function. Furthermore, cell types and their respective subtypes were annotated, leveraging canonical marker genes known for each cell type. To compute the ERS scores for individual cells, various methodologies such as AUCell and AddModuleScore were employed. The mean of these algorithms’ outputs was utilized as the final score to assess the ERS activity of each cell, ensuring a comprehensive evaluation of its cellular state.

### Cell–cell interaction

For analyzing cell–cell interactions, CellChat [22] software was implemented to synthesize gene expression data, focusing on hypothesized variations in cell communication modules. Utilizing CellChatDB as the primary ligand–receptor database, the standard CellChat procedure was followed. This approach enabled the deduction of cell type-specific interactions by pinpointing ligands or receptors that were predominantly expressed in certain cell groups. Enhanced interactions were determined based on the increased expression of these ligands or receptors.

### The selection of key genes

In the realm of key gene selection, the “findMarker” function played a pivotal role in identifying genes that exhibited marked differences between groups with high and low ERS scores. Genes demonstrating a fold change (FC) greater than 1.5 were deemed significant. Following this, a Spearman correlation analysis was conducted to pinpoint the top 150 genes that showed the most robust correlation with the ERS score. These genes, coupled with those significantly differentially expressed, formed the foundation for the development of subsequent analytical models.

### Building the high-performance ERS-associated signature (ERAS)

A univariate Cox regression analysis was employed to ascertain the impact of critical ERS genes on LUAD patient survival. To ensure the inclusion of significant variables, a *P*-value threshold of 0.05 was established. This step was instrumental in identifying genes with potential relevance to survival outcomes. Following this, the LASSO Cox regression [23] technique was utilized to streamline the pool of candidate genes. This method effectively refined the selection, leading to the formation of an optimal survival signature. The efficacy of the predictive model was evaluated using receiver operating characteristic (ROC) curves. In this assessment, an area under the curve (AUC) value surpassing 0.65 was indicative of superior predictive performance, confirming the model’s robustness in forecasting survival outcomes.

### Mutation landscape

GISTIC 2.0 analysis, accessible via GATK, was implemented to detect genomic alterations, specifically focusing on regions with frequent amplifications or deletions. Additionally, the “maftools” R package played a crucial role in determining tumor mutational burden (TMB), facilitating a deeper understanding of the genetic landscape of the tumors [24].

### Differences in the TME and drug inference

A suite of seven distinct immune infiltration algorithms was leveraged to conduct an exhaustive analysis of immune cell composition within various ERAS groups. Heatmaps were then effectively utilized to visually represent and highlight the nuanced variations in immune cell infiltration among these groups, thereby shedding light on the subtle differences in immune cell populations. The “estimate” R package [25] is employed to assess the relative values of immune cells, stromal cells, and tumor purity between high- and low-ERAS groups. Oncopredict [26] is utilized to evaluate the relative sensitivity of drugs between high- and low-ERAS groups, utilizing IC50 to assess drug sensitivity.

### Clinical specimen collection

We utilized the best website (https://rookieutopia.com/app_direct/BEST/) to explore the expression differences of model genes within ERAS between normal and tumor samples across multiple datasets [27]. The Medical Ethics Committee of Tianjin Medical University Cancer Institute and Hospital approved the collection of 10 paired LUAD tissue samples for this study. The primer sequences information is given in Table S1. Total RNA extraction from LUAD tissues was performed using Thermo Fisher Scientific’s TRIzol reagent, based in Waltham, MA, USA. Following the manufacturer’s instructions, cDNA synthesis was conducted using Thermo Fisher Scientific’s RevertAid™ First Strand cDNA Synthesis Kit. For quantitative real-time PCR (qRT-PCR) analysis, the StepOne Real-Time PCR system by Thermo Fisher Scientific was utilized, in conjunction with Takara Bio’s SYBR Green PCR kit, originating from Otsu, Japan. The relative gene expression was quantified using the 2^−△△CT^ method.

#### Statistical analysis

For all data processing, statistical analyses, and visualizations, R software (version 4.2.0) was the primary tool. Overall survival (OS) specific to each subtype was estimated and compared using the Kaplan–Meier method, with the log-rank test applied for validation. For continuous variables, comparisons between two groups were made using either the Wilcoxon test or *t*-test, depending on the data’s nature. Categorical variables were analyzed employing the Chi-squared test or Fisher’s exact test as appropriate. *P*-values underwent adjustment using the false discovery rate (FDR) method to enhance accuracy. Pearson correlation analysis was the chosen method for assessing relationships between variables. All *P*-values were derived from a two-tailed perspective, with *P* < 0.05 established as the criterion for statistical significance.

## Results

**Fig. 1:**
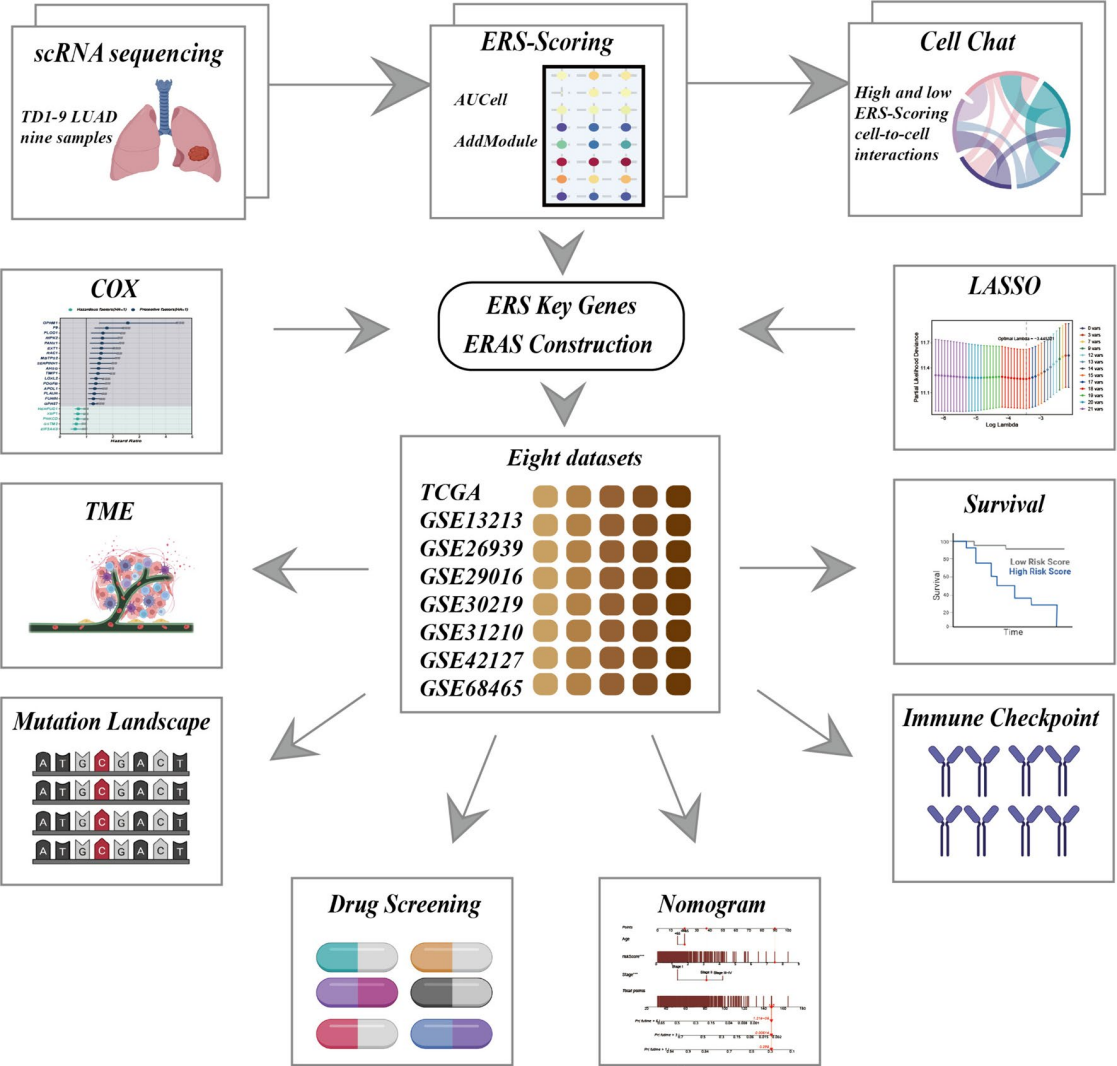
Experimental design flowchart

The schematic diagram depicting the workflow of this study is presented in Fig. 1.

### Single-cell sequencing data analysis

**Fig. 2:**
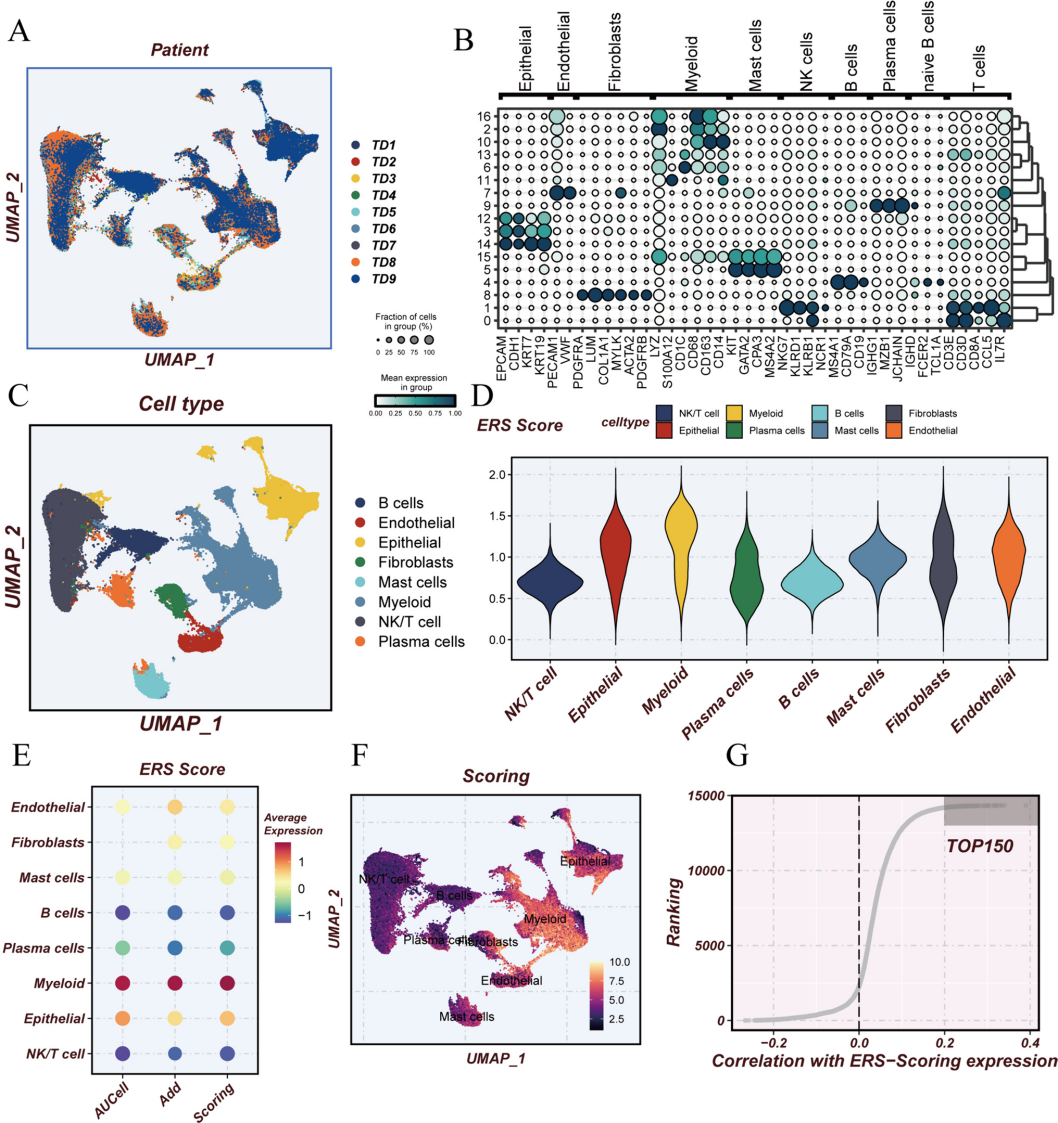
Cellular subpopulation annotation. **A** Sample distribution unaffected by batch effects. **B** Bubble plot illustrating representative marker genes. **C** UMAP plot demonstrating dimensionality reduction. **D** Violin plot displaying scoring patterns for each cell type. **E** Bubble plot comparing ERS scores in different cell types using two methods. **F** Heatmap offering visual representation of ERS scores across cell types. **G** Top 150 genes with the highest correlation to ERS

Initially, employing the UMAP algorithm, all samples were partitioned into nine distinct clusters (Fig. 2A). The average expression levels of specific gene markers for each cell type are depicted in Fig. 2B. Subsequently, based on specific gene markers, all cells were classified into eight different cell types (Fig. 2C). Figure 2D demonstrates the heterogeneity of ERS activity across different cell types, highlighting the varying roles of ERS within cells. Bubble plot further compares the ERS activity among the eight cell types using two different methods, revealing significantly higher expression of ERGs in myeloid cells compared to the other seven cell types (Fig. 2E). This finding is validated in the heatmap (Fig. 2F). Additionally, all genes were ranked based on their correlation with ERS, and the top 150 genes are depicted in the shaded area of Fig. 2G.

### Cellular communication

**Fig. 3:**
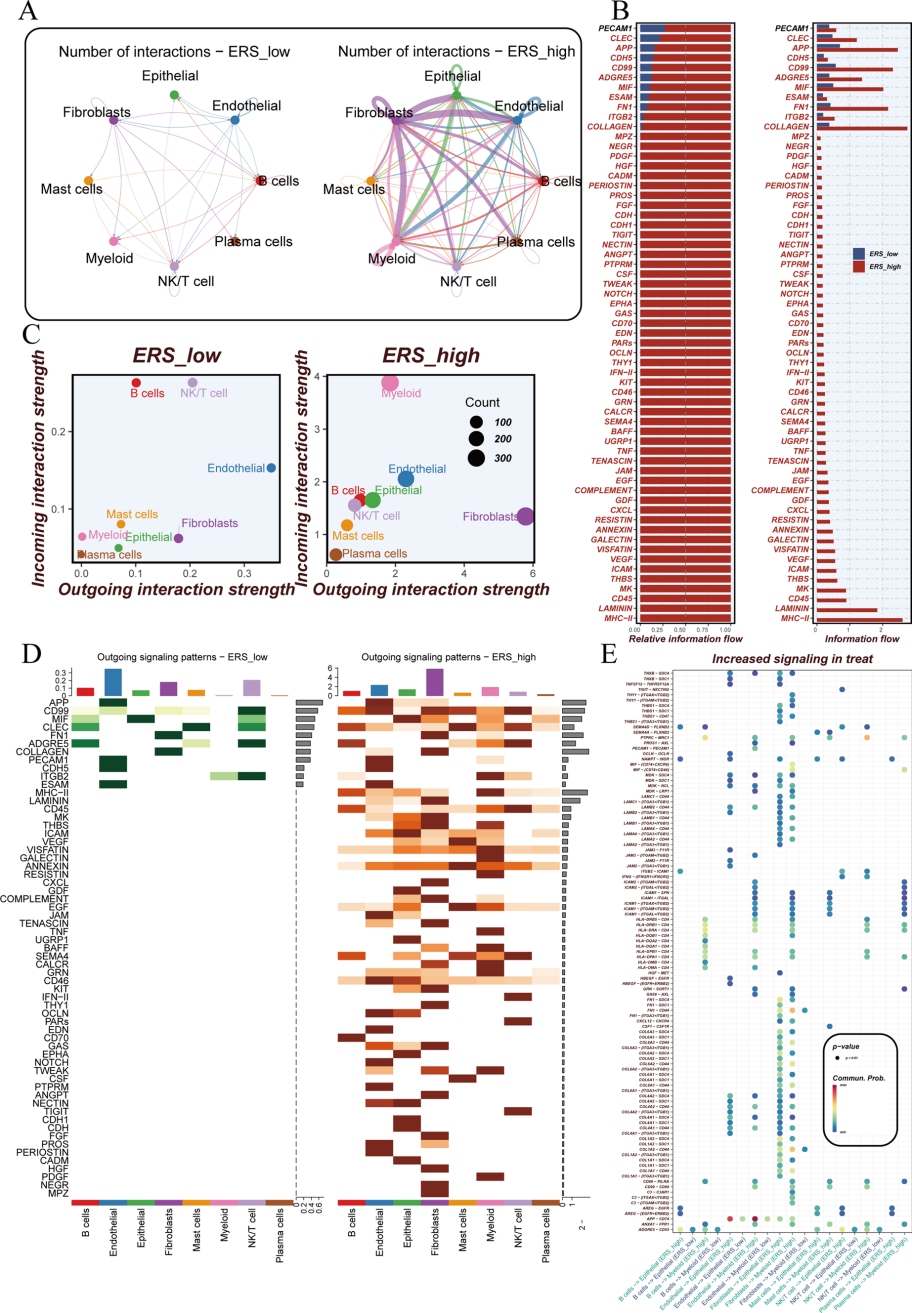
Intercellular communication among cells. **A** Signaling communication quantity between cells in ERS_High and ERS_Low groups. **B** Signaling pathway number and percentage in ERS_High and ERS_Low groups. **C** Differences in input and output signals between ERS_High and ERS_Low groups. **D** Differences in output signal patterns between ERS_High and ERS_Low groups. **E** Bubble plot illustrating the status of ligand–receptor pairs between the two groups

Based on the levels of ERS activity, all samples were divided into two groups: ERS_high and ERS_low. Figure 3A illustrates the intercellular communication among the eight cell types, clearly indicating a higher number of communication events in the ERS_high group compared to the ERS_low group. Similarly, the ERS_high group exhibits a significantly higher number and percentage of signaling pathways compared to the ERS_low group (Fig. 3B). Figure 3C compares the strength of input and output signals between the two groups, revealing lower intensities in the ERS_low group. Additionally, the ERS_high group demonstrates a more diverse pattern of signal outputs (Fig. 3D). Furthermore, there are notable differences in the number of ligand–receptor pairs between the two groups (Fig. 3E).

### Model construction and evaluation

**Fig. 4:**
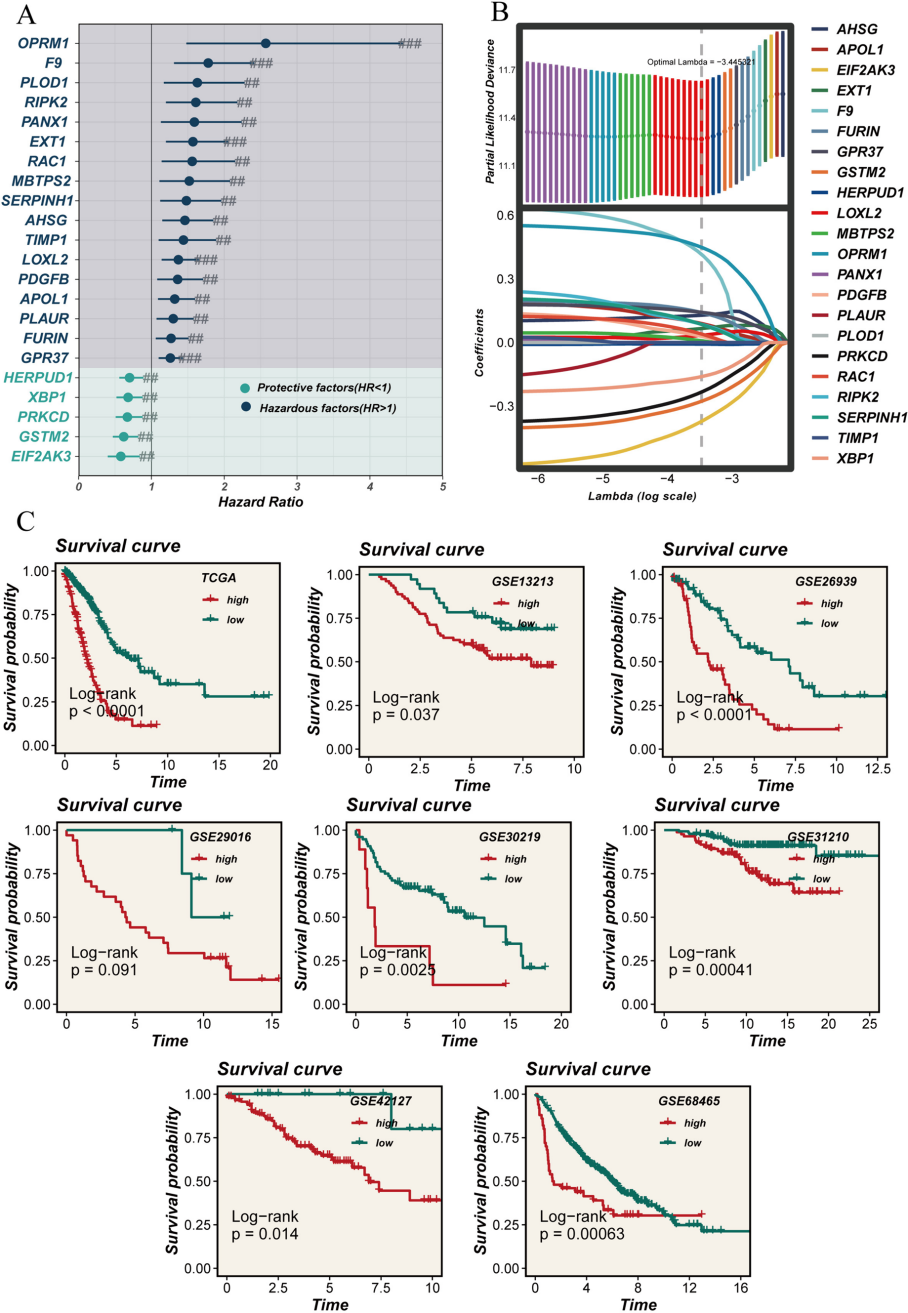
Modeling and survival analysis. **A** Results of univariate Cox analysis presented in a forest plot. **B** LASSO regression analysis. **C** Kaplan–Meier survival analysis in eight cohorts (TCGA_LUAD, GSE13213, GSE26939, GSE29016, GSE30219, GSE31210, GSE42127, and GSE68465)

**Fig. 5:**
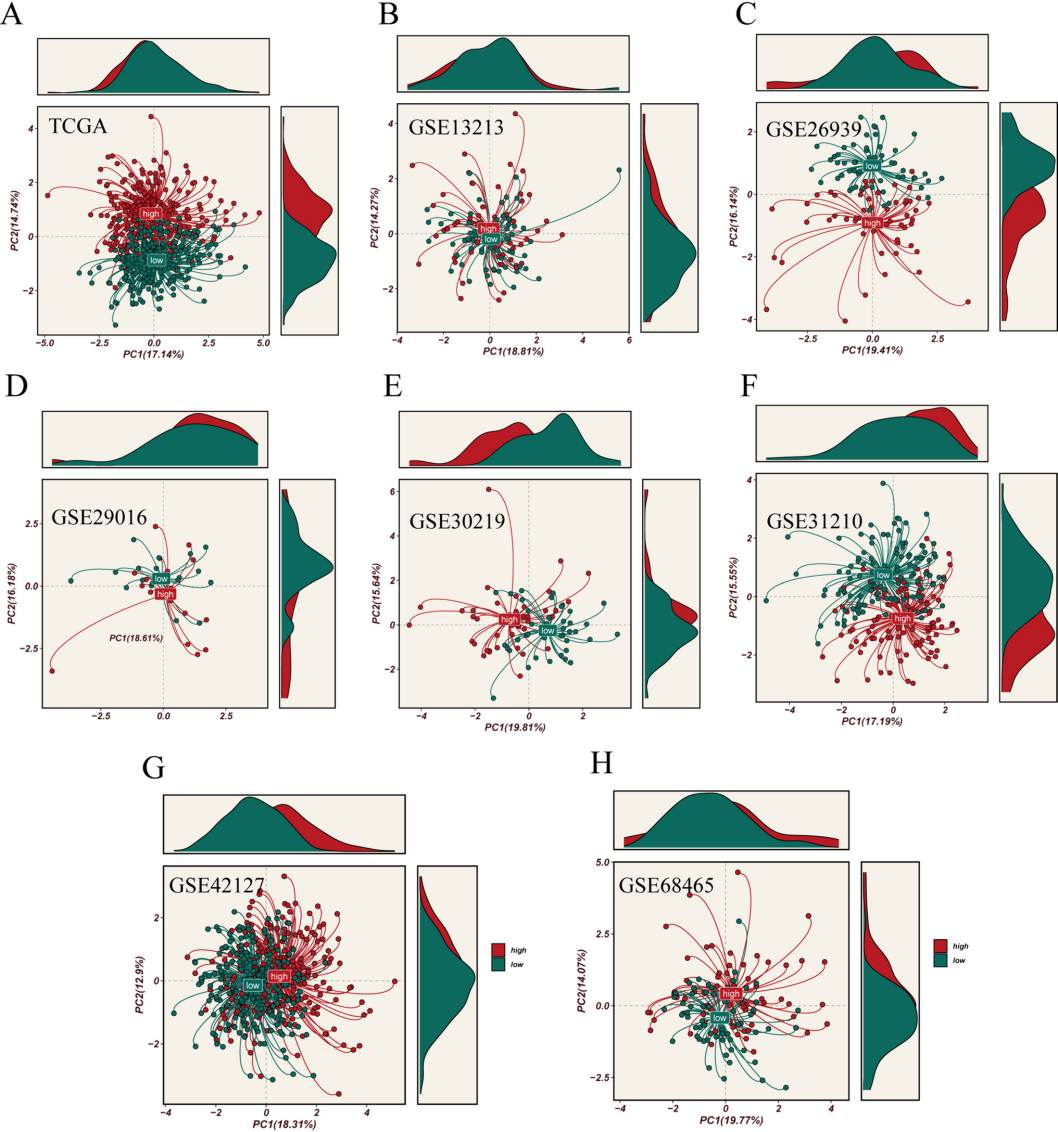
PCA analysis demonstrates effective sample discrimination based on scoring analysis in the TCGA, GSE13213, GSE26939, GSE29016, GSE30219, GSE31210, GSE42127, and GSE68465 datasets

To further elucidate ERGs influencing LUAD prognosis, we conducted univariate Cox regression analysis using differentially expressed genes between high and low ERS groups, along with the top 150 correlated genes. This analysis identified 22 prognostic genes, comprising 17 risk factors and five protective factors (Fig. 4A). Subsequently, we conducted LASSO regression analysis on these 22 genes in the TCGA cohort to select the optimal ERGs for constructing the prognostic model (Fig. 4B). Ultimately, we chose the 10 most influential ERGs to build the ERAS: F9, OPRM1, FURIN, SERPINH1, APOL1, GPR37, XBP1, PRKCD, GSTM2, and EIF2AK3 (Supplementary Fig. 1). According to the prognostic model, we calculated the ERAS score for each sample as follows: ERAS Score = ∑(Expi*coefi). Next, we divided all samples into high-ERAS and low-ERAS groups based on the median risk score. To evaluate the predictive ability of the model, we conducted KM survival analysis in the TCGA cohort, revealing significantly worse prognosis in the high-ERAS group compared to the low-ERAS group. This finding was consistent in the other six GEO cohorts (GSE13213, GSE26939, GSE29016, GSE30219, GSE31210, GSE42127, and GSE68465) (Fig. 4C). Furthermore, PCA analysis demonstrated effective separation of sample populations into two distinct clusters for the high- and low-ERAS groups, further confirming the accuracy and stability of the model (Fig. 5A–H).

### Independent prognostic analysis of the ERAS and construction of the nomogram

**Fig. 6:**
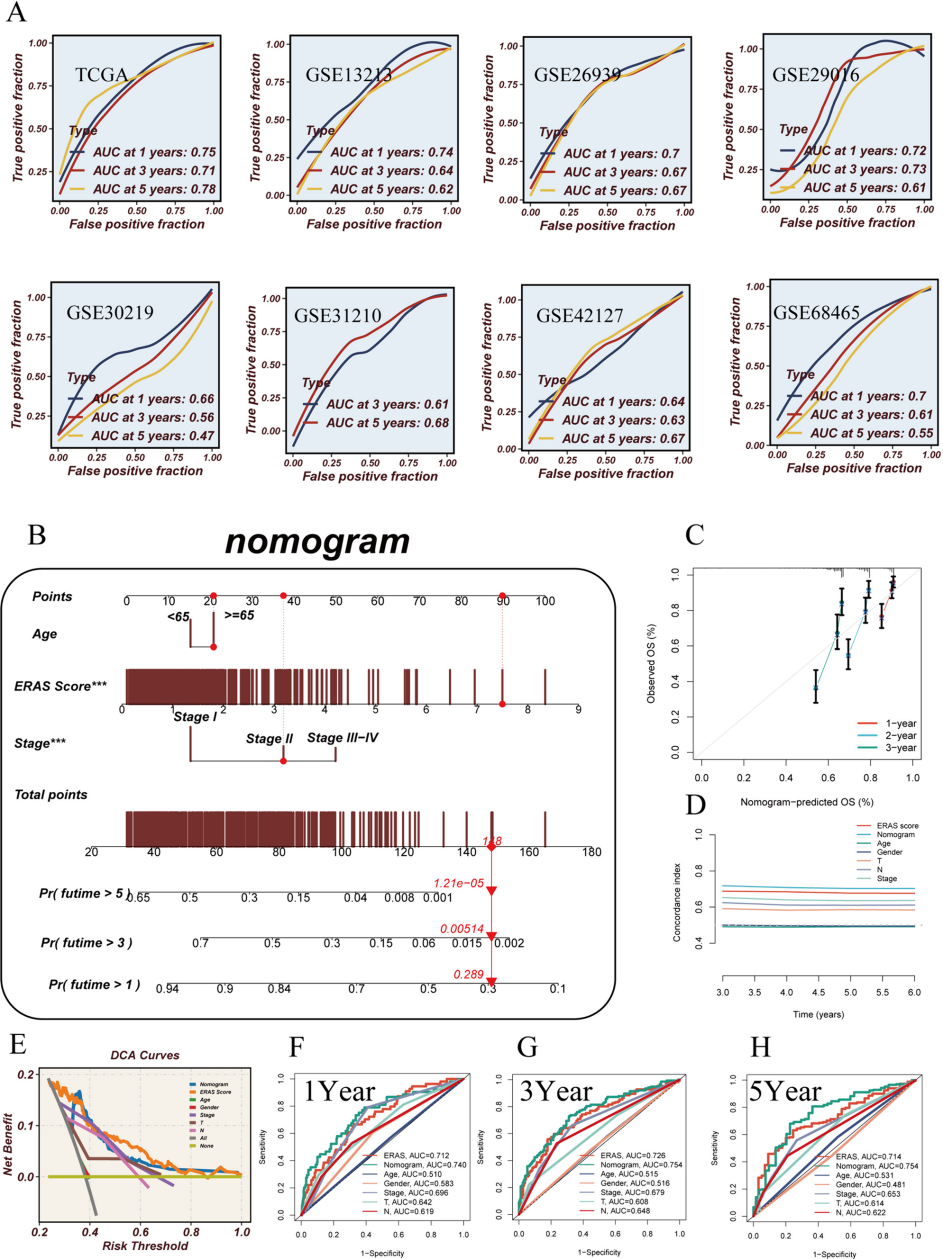
Independent validation of the model. **A** ROC curves illustrating the predictive performance of the model for 1-, 3-, and 5-year survival rates in different cohorts of LUAD patients. **B** Nomogram incorporating age, clinical stage, and risk score to predict 1-, 3-, and 5-year survival rates in LUAD patients. **C** Calibration curves. **D** Concordance index. **E** Decision curve analysis. **F**–**H** ROC curves demonstrating the predictive accuracy of ERAS score, nomogram, and other clinical features for 1-, 3-, and 5-year survival rates in LUAD patients

The AUC values for predicting 1-year, 3-year, and 5-year overall survival were also high, indicating good predictive performance of the model (Fig. 6A). To validate whether the predictive model is an independent prognostic factor for LUAD patients, we conducted a multivariable Cox regression analysis to evaluate the risk score and other clinical features. Our results demonstrated that age, stage, and risk score are independent prognostic indicators for LUAD. Based on these findings, we constructed a nomogram model incorporating the risk score and two additional clinical factors to predict the 1-year, 3-year, and 5-year overall survival of LUAD patients (Fig. 6B). Decision curve analysis, concordance index, and calibration curve analyses all indicated that the nomogram outperforms other clinical indicators in terms of accuracy and consistency in predicting patient prognosis. Therefore, it serves as a clinically valuable decision-making tool (Fig. 6C–E). Additionally, the ROC curve demonstrated that the nomogram model exhibits better predictive accuracy compared to other indicators (AUC at 1 year, 3 years, and 5 years: 0.740, 0.754, and 0.754) (Fig. 6F–H).

### Immune infiltration analysis

**Fig. 7:**
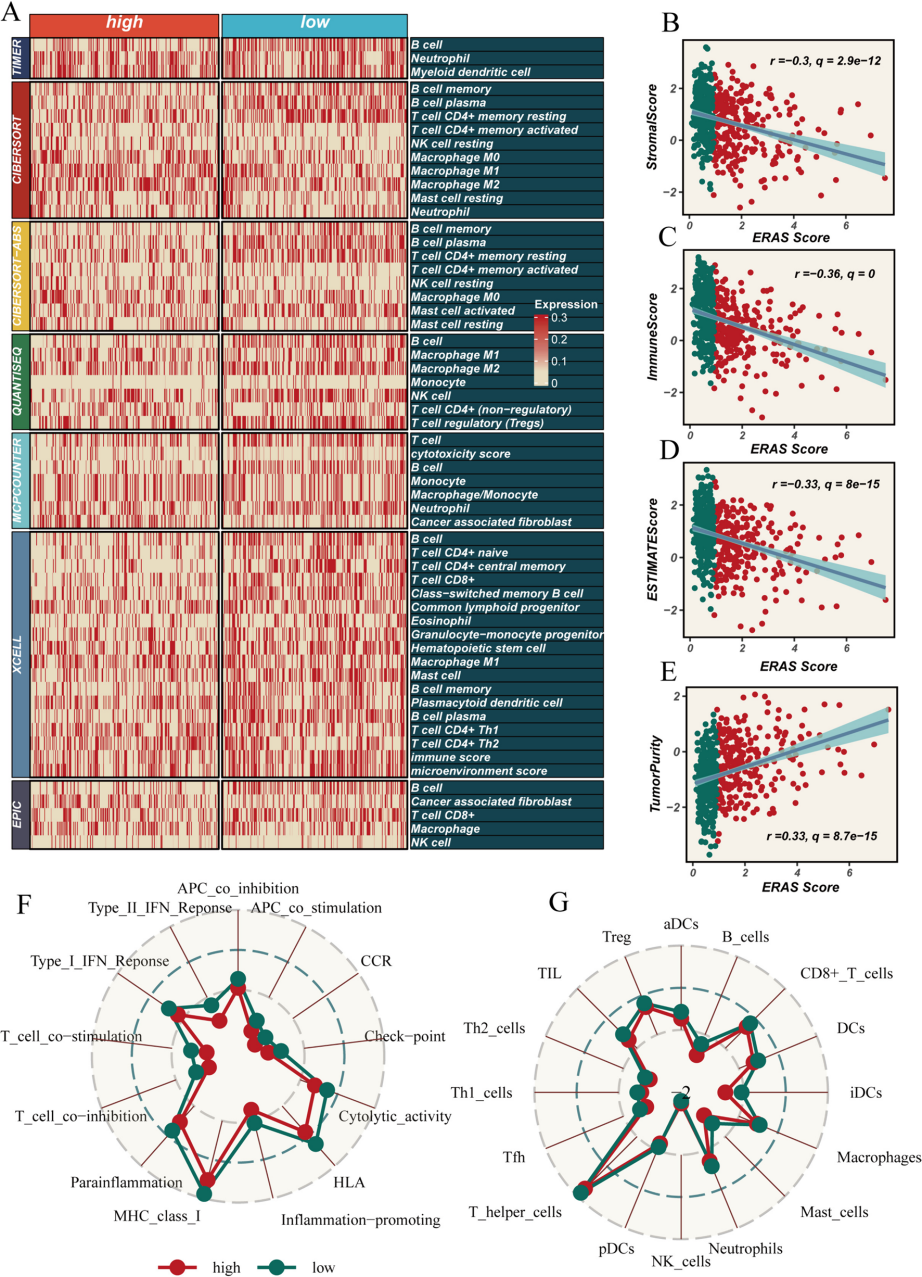
Analysis of immune infiltration. **A** Heatmap illustrating the immune infiltration patterns in the high- and low-ERAS groups. **B**–**E** Scatter plots displaying the correlation between ERAS score and stromal score, immune score, ESTIMATE score, and tumor purity. **F**, **G** Comparison of immune cell infiltration and immune-related pathway differences between the two groups

To investigate the role of immune cells in tumors, we employed seven methods for immune evaluation. The heatmap presents the immune infiltration status of the high- and low-risk groups. Although the differences are not statistically significant, it can be observed that the level of immune infiltration in the low-ERAS group is slightly higher than that in the high-ERAS group (Fig. 7A). Correlation analysis revealed a negative correlation between risk score and stromal score, immune score, and ESTIMATE score, while a positive correlation was observed with tumor purity (Fig. 7B–E). Furthermore, we assessed the degree of immune cell infiltration and immune-related pathways using the ssGSEA method. The results indicated that the low-ERAS group displayed higher levels of immune cell infiltration across all cell types. Additionally, the low-ERAS group exhibited stronger activity in most immune-related pathways compared to the high-ERAS group, such as Type_I IFN response, HLA, and Parainflammation (Fig. 7F and G). These findings suggest that the low-risk group demonstrates an overall higher immune status and immunogenicity.

### Tumor mutation burden (TMB)

**Fig. 8:**
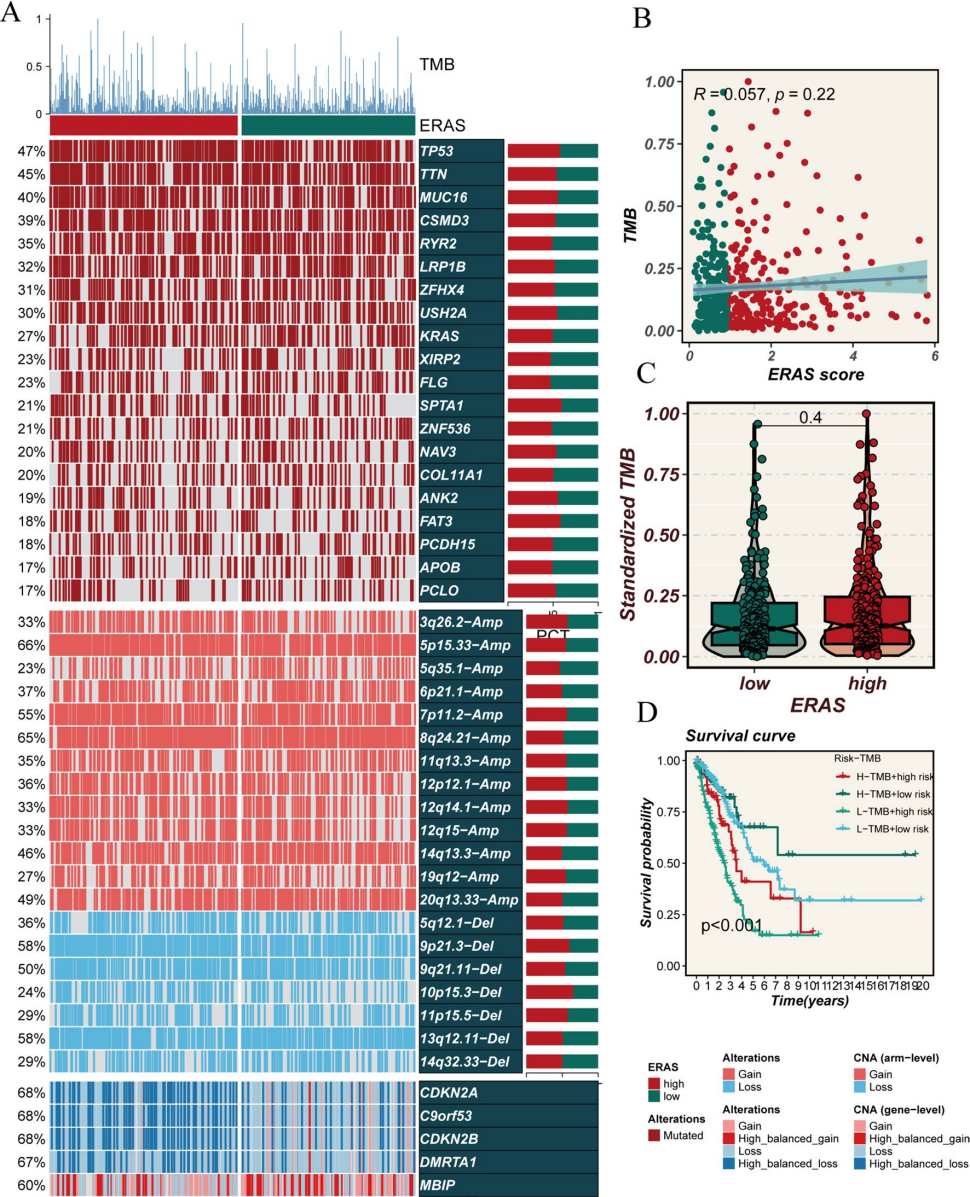
Analysis of mutation burden. **A** Heatmap illustrating the differential TMB between the high- and low-ERAS groups. **B** Scatter plots demonstrating the correlation between ERAS score and TMB. **C** Comparison of TMB between the high- and low-ERAS groups. **D** Kaplan–Meier survival curves displaying the prognostic differences among four subgroups (High-ERAS and High-TMB, High-ERAS and Low-TMB, Low-ERAS and High-TMB, Low-ERAS and Low-TMB)

The heatmap illustrates the distribution of TMB in the high- and low-ERAS groups (Fig. 8A). Although the difference between the two groups is not statistically significant, the high-ERAS group tends to have a higher overall TMB compared to the low-ERAS group (Fig. 8C). Furthermore, there is no clear correlation between risk score and TMB (Fig. 8B). Subsequent subgroup analysis revealed that the prognosis is poorest in the low-TMB + high-ERAS group (Fig. 8D).

### Drug sensitivity analysis

**Fig. 9:**
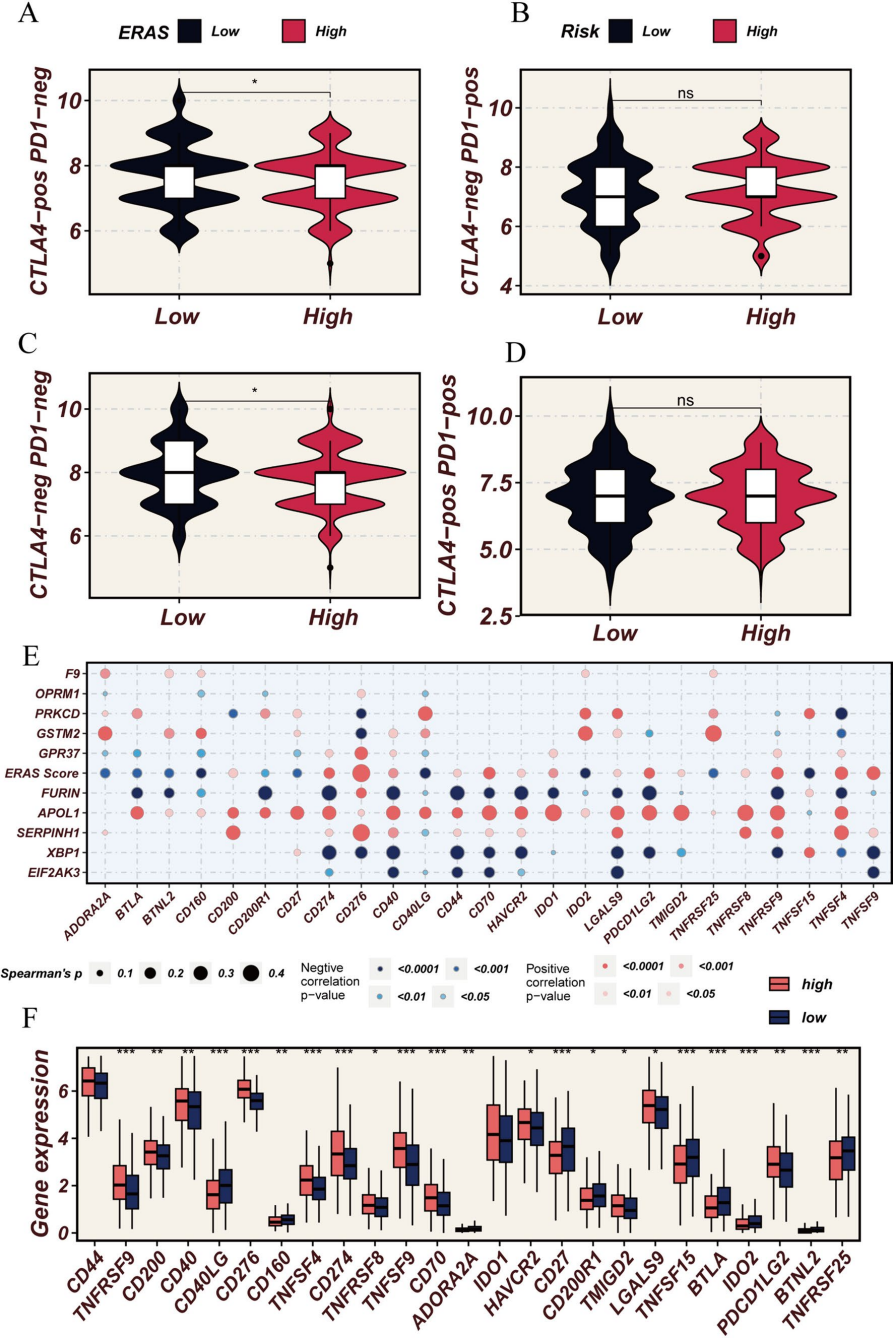
Immune checkpoints. **A**–**D** Comparison of immune prognostic score (IPS) between high- and low-ERAS groups to infer the receptivity to CTLA-4 and PD1 treatment. **E** Bubble plot illustrating the associations between ERAS score, model genes, and common immune checkpoints. **F** Boxplot comparing the expression differences of common immune checkpoints between the high- and low-ERAS groups

**Fig. 10:**
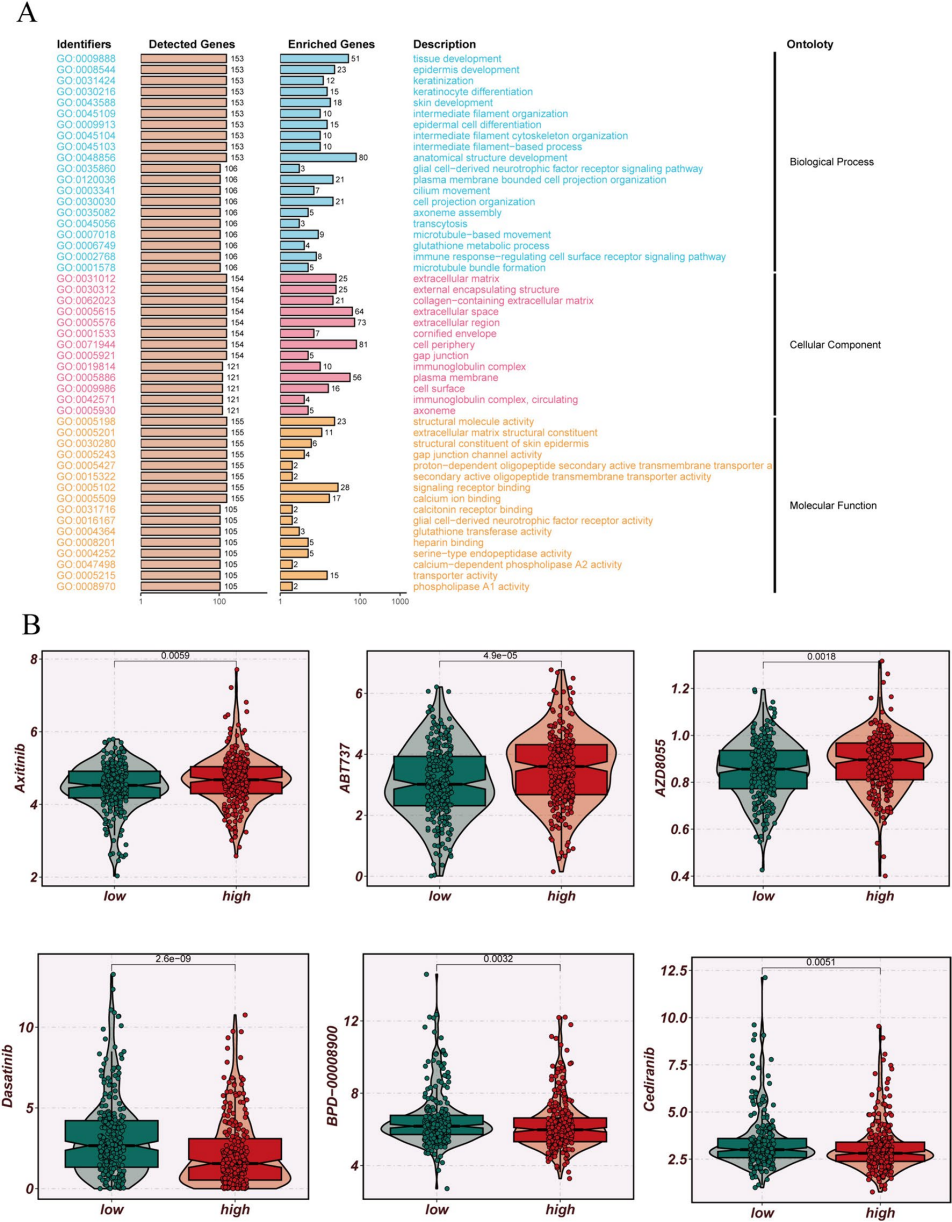
Enrichment analysis and drug sensitivity testing. **A** GO enrichment analysis. **B** Boxplot illustrating the sensitivity of the high- and low-ERAS groups to common chemotherapeutic drugs

We observed that patients in the low-ERAS group demonstrate elevated immunophenoscores (IPS) following CTLA-4 immune therapy. This implies that individuals in the low-ERAS group might exhibit heightened responsiveness to immune checkpoint inhibitors (ICIs), potentially yielding greater therapeutic benefits (Fig. 9A–D). The bubble plot illustrates the correlation between ERAS score, model genes, and common immune checkpoints (Fig. 9E). Additionally, differential analysis of immune checkpoints revealed significantly increased expression levels of CD40LG, CD160, CD27, and BTLA in the low-risk group compared to the high-risk group (Fig. 9F). To further investigate its underlying biological mechanisms, we conducted GSEA analysis. GO enrichment analysis revealed that ERS-related genes are primarily enriched in pathways such as tissue development, extracellular region, anatomical structure development, and cell periphery (Fig. 10A). Furthermore, we investigated the association between ERAS score and patient response to chemotherapy drugs. Axitinib, ABT737, and AZD8055 were found to potentially exhibit increased efficacy in the low-ERAS group, whereas Dasatinib, BPD-00008900, and Cediranib showed heightened sensitivity in the high-ERAS group (Fig. 10B). Axitinib is a tyrosine kinase inhibitor (TKI) primarily targeting vascular endothelial growth factor receptors (VEGFRs), demonstrating efficacy in LUAD treatment by inhibiting angiogenesis. ABT737 is a BH3 mimetic compound that induces apoptosis by targeting B-cell lymphoma 2 (Bcl-2) family proteins, potentially offering therapeutic benefits in LUAD characterized by dysregulated apoptosis pathways. AZD8055 is an mTOR inhibitor that can suppress the PI3K/AKT/mTOR signaling pathway, crucial in LUAD progression and resistance to therapy. On the other hand, Dasatinib is a potent multi-kinase inhibitor targeting Src family kinases and other tyrosine kinases, showing promise in LUAD therapy, especially in cases where Src activation plays a role in tumor progression. BPD-00008900 is an anti-angiogenic agent with potential in inhibiting tumor growth and metastasis, offering avenues for targeted therapy in LUAD. Cediranib is another VEGFR inhibitor that can suppress angiogenesis, potentially hindering tumor growth and metastasis in LUAD. Understanding the differential responses of these drugs based on ERAS scores provides valuable insights into personalized treatment strategies for LUAD patients, potentially optimizing therapeutic outcomes and minimizing adverse effects.

### Enrichment analysis

Enrichment analysis focusing on individual model genes indicated that genes associated with GPR37 are predominantly enriched in pathways related to intracellular anatomical structure, binding, and protein binding. Genes associated with SERPINH1, on the other hand, are primarily enriched in pathways related to binding, protein binding, cytoplasm, and endomembrane system (Supplementary Fig. 2A and B).

### Experimental validation

**Fig. 11:**
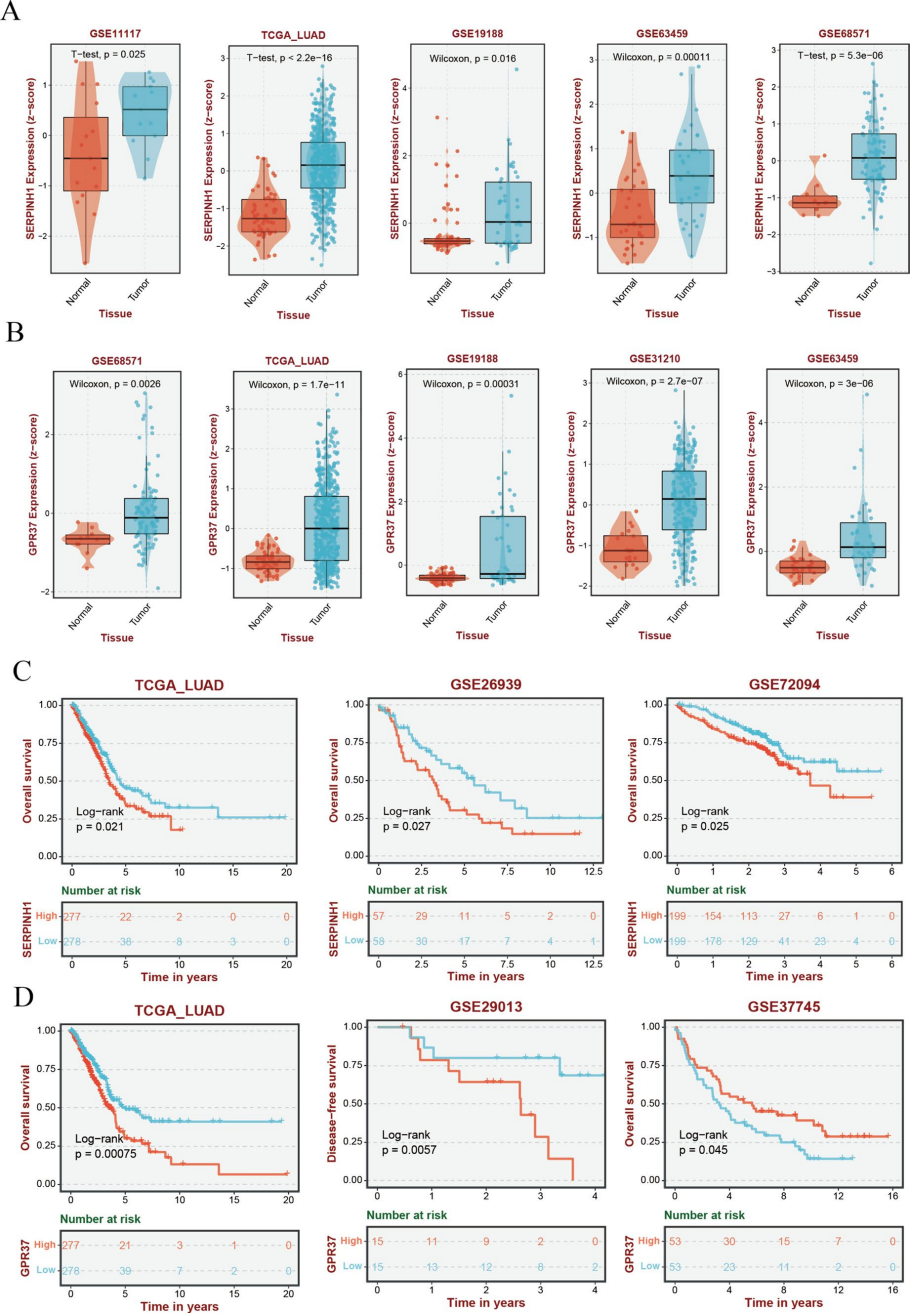
Validation of model genes. (A) Comparison of SERPINH1 gene expression differences between tumor and normal tissues in five cohorts (TCGA_LUAD, GSE11117, GSE19188, GSE63459, and GSE68571). **B** Comparison of GPR37 gene expression differences between tumor and normal tissues in five cohorts (TCGA_LUAD, GSE19188, GSE63459, GSE68571, and GSE31210). **C** Kaplan–Meier survival analysis for SERPINH1 in three cohorts (TCGA_LUAD, GSE26939, and GSE72094). **D** Kaplan–Meier survival analysis for GPR37 in three cohorts (TCGA_LUAD, GSE29013, and GSE37745)

To enhance the credibility of our research findings, we performed preliminary experimental validations. In TCGA, significant differences were observed in the expression levels of SERPINH1 and GPR37 between normal tissues and LUAD tumor tissues. This result was further validated in cohorts such as GSE11117, GSE19188, GSE63459, GSE68571, and GSE31210 (Fig. 11A and B). qPCR experiments also demonstrated markedly upregulated expression of SERPINH1 and GPR37 in tumor tissues (Supplementary Fig. 3A and B). Additionally, survival analysis of SERPINH1 (TCGA_LUAD, GSE26939, and GSE72094) and GPR37 (TCGA_LUAD, GSE29013, and GSE37745) in multiple cohorts revealed a poorer prognosis in the high-expression groups (Fig. 11C and D). These results suggest that SERPINH1 and GPR37 play a tumor-promoting role in LUAD, validating their detrimental effects in the ERAS model.

## Discussion

LC, the leading cause of cancer-related mortality worldwide [28], poses a significant threat to human health. Currently, the treatment selection for many lung cancer patients relies heavily on TNM staging. However, due to tumor heterogeneity, patients in the same TNM stage can exhibit varying prognoses, indicating the need for updated patient classification methods [29, 30]. The implementation of precision therapy for lung cancer and the effective reduction of mortality rates pose urgent public health challenges [31, 32]. In recent years, researchers have increasingly focused on predictive indicators at the cellular and molecular levels to explore personalized prediction and treatment approaches, leading to some notable achievements [33–35].

Increasing evidence suggests that ERS has a significant impact on tumor cell survival [11, 36]. Activation of ERS can induce tumor cell apoptosis or inhibit tumor cell proliferation by causing cell cycle arrest. However, dysregulation of ERS can also promote tumor cell invasion and metastasis, thereby enhancing tumor malignancy [37, 38]. This implies that precise modulation of ERS function holds the potential for systematic clearance of tumors. It is worth noting that there is currently limited availability of LUAD prognostic models based on ERS-related genes, and the role of ERS in LUAD remains unclear.

In this study, we utilized LASSO regression analysis to identify 10 ERGs (F9, OPRM1, FURIN, SERPINH1, APOL1, GPR37, XBP1, PRKCD, GSTM2, and EIF2AK3) and constructed a LUAD prognostic model called ERAS based on these genes. Subsequently, we performed KM survival analysis in eight LUAD cohorts (TCGA_LUAD, GSE13213, GSE26939, GSE29016, GSE30219, GSE31210, GSE42127, and GSE68465) to validate the effective predictive capability of ERAS for LUAD prognosis. Furthermore, immune infiltration analysis and tumor mutation burden analysis provided additional validation of ERAS’s discriminative ability. To gain further insights into the role of ERS in LUAD, we conducted enrichment analysis, which revealed that ERGs are primarily enriched in pathways such as tissue development, extracellular region, anatomical structure development, and cell periphery. Through drug sensitivity analysis, we identified several chemotherapeutic drugs that exhibit efficacy in the high- and low-ERAS groups. Finally, preliminary qPCR experiments were conducted to validate the significant role of SERPINH1 and GPR37 in LUAD prognosis.

The protein encoded by the SERPINH1 gene belongs to the H family of the serpin superfamily, also known as heat shock protein 47 (HSP47). HSP47 primarily resides in the ER, where it acts as a chaperone protein during protein folding and repair processes, effectively preventing abnormal folding and aggregation [39]. The previous studies have indicated a close association between SERPINH1 and cancer development, as it can facilitate cancer growth and invasion by modulating the extracellular matrix [40, 41]. Furthermore, SERPINH1 has been identified as a potential molecular marker and therapeutic target in various malignant tumors [42–44]. Mortezapour et al. found that SERPINH1 is overexpressed in colorectal cancer, and miR-940 targeting SERPINH1 could serve as a potential biomarker for colorectal cancer [45]. Xia et al. discovered that SERPINH1 may promote proliferation and migration in osteosarcoma, and their SERPINH1-related model successfully predicted the immunological characteristics and response to immunotherapy in osteosarcoma patients [46]. In this study, the overexpression of SERPINH1 in LUAD tissues is often indicative of a worse prognosis. Compared to SERPINH1, the research on GPR37 is currently limited. It is known that GPR37 encodes a G protein-coupled receptor (GPCR) that may play an important role in neurodevelopment and neurodegenerative diseases [47, 48]. In recent years, some researchers have discovered that GPR37 is significantly upregulated in tumors and has a certain impact on tumor prognosis [49]. Xie et al. found that GPR37 is overexpressed and associated with poor prognosis in LUAD, and the mechanism may involve the interaction of GPR37 with CDK6 to induce cell cycle arrest, thereby promoting tumor progression [50]. These findings are consistent with the results of this study.

While this study offers valuable insights, several limitations need acknowledgment. Firstly, the underlying mechanisms behind the findings were not thoroughly investigated. Secondly, the restricted availability of data on LUAD patients in public databases led to a relatively small sample size and a lack of essential information, potentially compromising the representativeness of the study results. Thirdly, further in vivo and in vitro experiments are necessary for validation.

In conclusion, we have developed a signature based on 10 ERS-related genes capable of effectively predicting the prognosis of LUAD patients. Furthermore, through this model, we have identified several chemotherapeutic drugs exhibiting efficacy in LUAD patients. These ERS-based findings contribute to our understanding of LUAD biology and offer new perspectives for precision treatment approaches.

## Supplementary Information

Below is the link to the electronic supplementary material.

Supplementary file1 Supplementary Fig. 1. Coefficients of Model Genes. (TIF 938 KB)

Supplementary file2 Supplementary Fig. 2. GO Enrichment Analysis for SERPINH1 and GPR37. (TIF 2616 KB)

Supplementary file3 Supplementary Fig. 3. qPCR Experiment. (A) Differential Expression of GPR37 Between Tumor and Normal Tissues in TCGA_LUAD. Relative Expression of GPR37 Gene in 10 Pairs of Cancer and Paracancer Samples, Respectively. (B) Differential Expression of SERPINH1 Between Tumor and Normal Tissues in TCGA_LUAD. Relative Expression of SERPINH1 Gene in 10 Pairs of Cancer and Paracancer Samples, Respectively. (TIF 1195 KB)

Supplementary file4 (XLSX 10 KB)

## Data Availability

The datasets analyzed in the current study are available in the TCGA repository (http://cancergenome.nih.gov/), and GEO (https://www.ncbi.nlm.nih.gov/geo/).
