# Supplementary figures and images for: Deciphering the prognostic role of endoplasmic reticulum stress in lung adenocarcinoma: integrating prognostic prediction and immunotherapy strategies
Source: Clin Exp Med. 2024 Jul 25;24(1):169. doi: 10.1007/s10238-024-01439-4 (PMC11272744; doi:10.1007/s10238-024-01439-4)

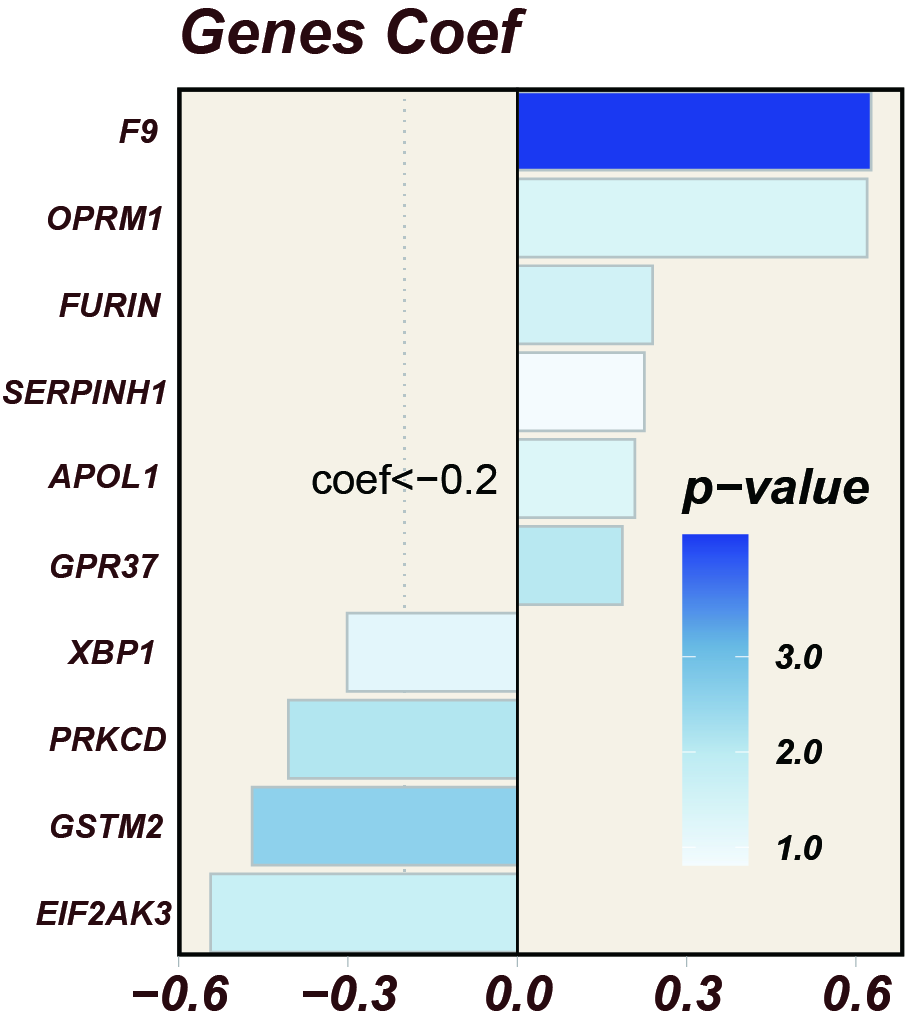

Supplement: Supplementary file 1 — Supplementary file1 Supplementary Fig. 1. Coefficients of Model Genes. (TIF 938 KB) [file 10238_2024_1439_MOESM1_ESM.tif]

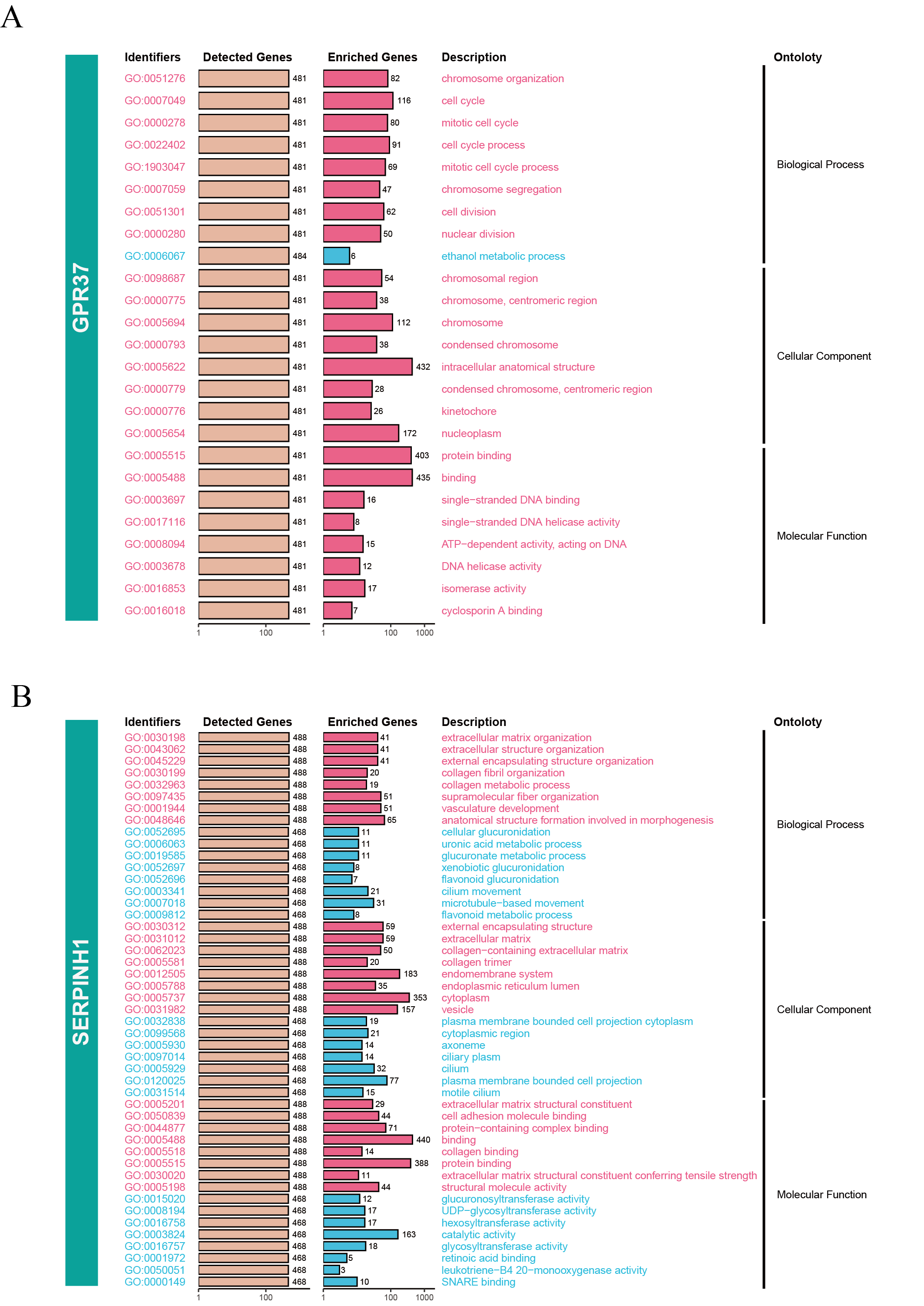

Supplement: Supplementary file 2 — Supplementary file2 Supplementary Fig. 2. GO Enrichment Analysis for SERPINH1 and GPR37. (TIF 2616 KB) [file 10238_2024_1439_MOESM2_ESM.tif]

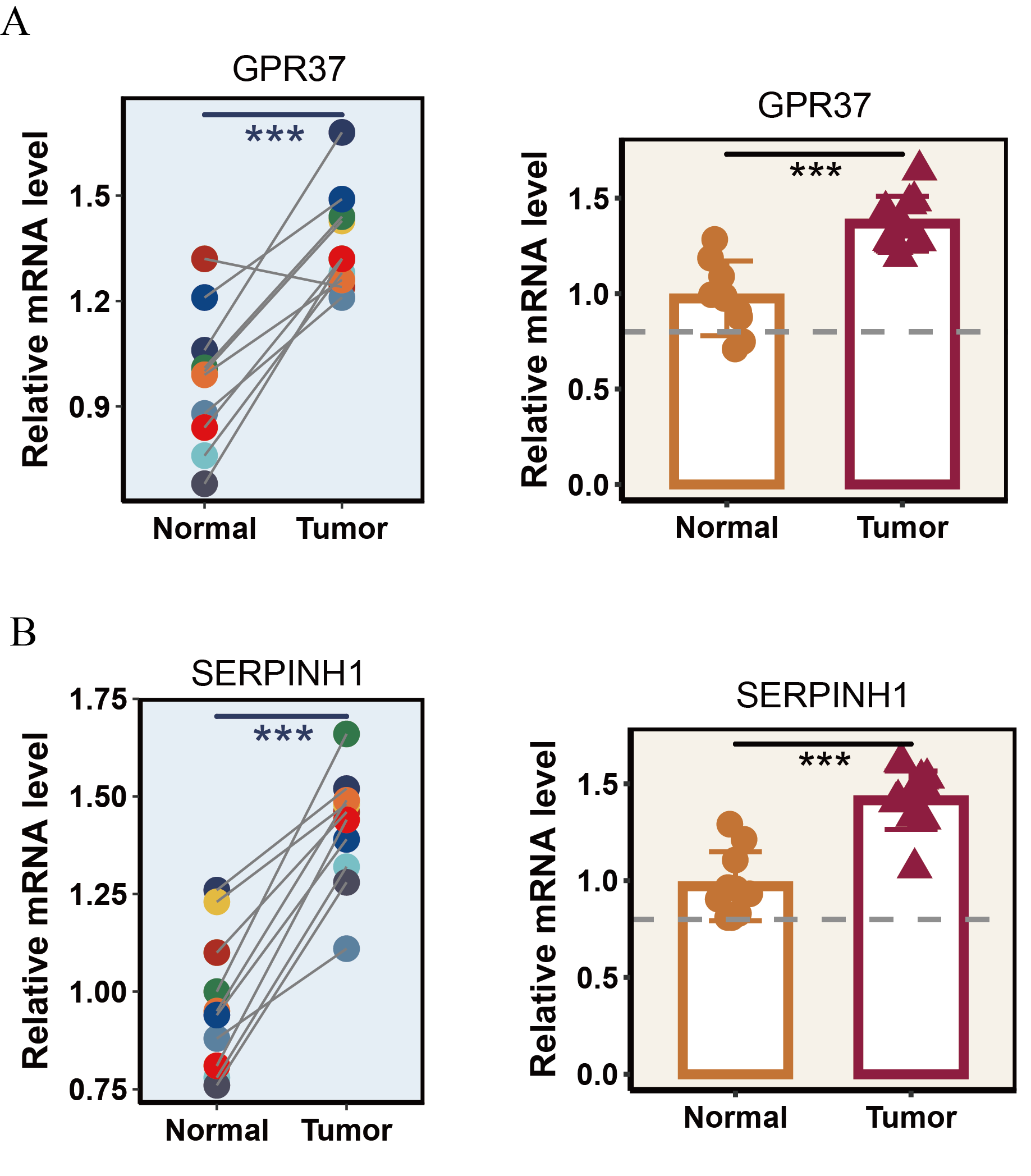

Supplement: Supplementary file 3 — Supplementary file3 Supplementary Fig. 3. qPCR Experiment. (A) Differential Expression of GPR37 Between Tumor and Normal Tissues in TCGA_LUAD. Relative Expression of GPR37 Gene in 10 Pairs of Cancer and Paracancer Samples, Respectively. (B) Differential Expression of SERPINH1 Between Tumor and Normal Tissues in TCGA_LUAD. Relative Expression of SERPINH1 Gene in 10 Pairs of Cancer and Paracancer Samples, Respectively. (TIF 1195 KB) [file 10238_2024_1439_MOESM3_ESM.tif]
